# Antibiotic Outcomes of Enterococcal Urinary Tract Infections: A Retrospective Analysis from Saudi Arabia and Oman

**DOI:** 10.3390/pathogens15030250

**Published:** 2026-02-26

**Authors:** Abrar K. Thabit, Juhaina S. Al-Maqbali, Khaled F. Alharthi, Salem M. Baotab, Abdullah M. Bankhar, Rayyan M. Wali, Mohammed O. Alzahrani, Asiya K. Alharthi, Taqwa M. Alhamsaidi, Ibrahim Al Busaidi, Ahmad J. Mahrous, Jimmy Jose

**Affiliations:** 1 Department of Pharmacy Practice, Faculty of Pharmacy, King Abdulaziz University, Jeddah 22254, Saudi Arabia; 2 Department of Pharmacology and Clinical Pharmacy, College of Medicine and Health Science, Sultan Qaboos University, Muscat 123, Oman; 3 Department of Pharmacy, Sultan Qaboos University Hospital, University Medical City, Muscat 123, Oman; 4 Faculty of Pharmacy, King Abdulaziz University, Jeddah 22254, Saudi Arabia; 5 School of Pharmacy, University of Nizwa, Nizwa 616, Oman; 6 Department of Medicine, Sultan Qaboos University Hospital, University Medical City, Muscat 123, Oman; 7 Department of Pharmaceutical Practice, College of Pharmacy, Umm Al-Qura University, Makkah 21955, Saudi Arabia

**Keywords:** antibiotics, *Enterococci*, *Enterococcus faecalis*, *Enterococcus faecium*, outcomes, urinary tract infection

## Abstract

Background: *Enterococcus* species are involved in urinary tract infections (UTIs), and they are known to be intrinsically resistant to certain antibiotics. We aimed to investigate the clinical characteristics and treatment outcomes of enterococcal UTIs in three hospitals in Saudi Arabia and Oman. Methods: A retrospective cohort study was conducted on adults with clinically and microbiologically confirmed enterococcal UTI based on urinary symptoms and a urine culture of ≥100,000 CFU/mL, who received an antibiotic active against the pathogen. The primary endpoint was clinical cure. Secondary endpoints included microbiological cure, length of stay (LOS), in-hospital mortality, and recurrence. Results: *E. faecalis* and *E. faecium* were isolated from 188 (67.1%) and 92 (32.9%), respectively, of 280 included patients. Ampicillin/amoxicillin (25%) and vancomycin (22.1%) were the most-used antibiotics. Compared with *E. faecium*, *E. faecalis* was associated with higher clinical cure rates (75% vs. 57.6%; *p* = 0.003), lower in-hospital mortality (15.7% vs. 38.5%; *p* < 0.0001), and shorter LOS (12.5 vs. 25 days; *p* < 0.0001). No difference in recurrence was observed. Ciprofloxacin was associated with high odds of clinical cure (OR, 4.28; 95% CI, 1.18–15.56). Conversely, the recent cancer chemotherapy and growth of *Enterococcus* at another site were associated with lower odds of clinical cure. Urinary catheter removal was associated with lower recurrence odds (OR, 0.48; 95% CI, 0.24–0.98). Conclusions: This study highlights the clinical challenges posed by enterococcal UTIs, particularly by *E. faecium*. Ciprofloxacin remains an effective option, particularly against *E. faecalis*. Patients with advanced age, critical illness, complicated infections, and liver disease, as well as patients on hemodialysis, require close monitoring to improve outcomes.

## 1. Introduction

*Enterococci* are Gram-positive chain-like cocciform bacteria that are normally present in the gastrointestinal tract and function commensally with humans [1,2]. However, they are associated with a wide variety of infections, such as urinary tract infections (UTIs), bacteremia, and endocarditis, which often require hospitalization [1,2]. Two species cause the majority of enterococcal infections: *Enterococcus faecalis* and *E. faecium* [3].

*Enterococci* have developed several mechanisms to evade the effects of commonly used antibiotics. As the resistance of *Enterococci* to commonly used antibiotics (such as vancomycin) is on the rise, alternative antibiotics are being considered. Treatment options for UTIs caused by *Enterococci* include oral agents, such as amoxicillin and nitrofurantoin, as well as parenteral agents, such as ampicillin, vancomycin, teicoplanin, quinupristin/dalfopristin, gentamicin, linezolid, and daptomycin [4]. However, these options are not always feasible due to patient factors (e.g., potential adverse effects that may aggravate a patient’s condition, such as nephrotoxicity with vancomycin and thrombocytopenia with linezolid), cost, and local unavailability. *Enterococci* are known to be intrinsically resistant to some antimicrobials, such as cephalosporins, clindamycin, and trimethoprim/sulfamethoxazole [4]. A plastic genome allows these two significant species of *Enterococci* to readily acquire resistance to more antibiotics, such as high-level aminoglycoside resistance, high-level ampicillin resistance, and vancomycin resistance, either through mutation or by horizontal transfer of genetic elements conferring resistance determinants [3]. Furthermore, *Enterococci* can acquire resistance to other antimicrobials after exposure or through the transfer of resistance genes from the same or other bacterial species. Such acquired resistance can be developed against vancomycin, β-lactams, aminoglycosides, erythromycin, fluoroquinolones, tetracycline, rifampin, nitrofurantoin, and chloramphenicol [5,6]. Enterococcal infections may be difficult to treat and present ongoing therapeutic challenges to clinicians worldwide because of their broad spectrum of intrinsic resistance and tolerance to the bactericidal activity of many agents, combined with their prodigious ability to acquire resistance to available antibiotics [3].

A previous study from Saudi Arabia evaluated the clinical characteristics of patients with enterococcal UTIs, finding that a non-intensive care unit (ICU) admission, recent exposure to antibiotics within the last three months, and the presence of a urinary catheter were prevalent among the patients [7]. Furthermore, despite a lower prevalence of *E. faecium* infections compared with *E. faecalis* infections, a comparison between the two species in terms of clinical outcomes revealed that infections with *E. faecium* were associated with higher levels of inflammatory markers (namely C-reactive protein), higher mortality, and longer hospital stays, due to variations in virulence factors or increased rates of antibiotic resistance in *E. faecium* as opposed to *E. faecalis* [7]. In terms of comparing antibiotic therapy outcomes, very few studies compared treatment options for enterococcal UTIs. Nonetheless, one retrospective cohort study compared the effectiveness of linezolid and non-linezolid antibiotics (including nitrofurantoin) in the treatment of UTIs caused by vancomycin-resistant *Enterococci* (VRE) and found no significant difference in clinical cure rates between the two groups [8]. An in vitro study found that the susceptibility rates of *Enterococcus* spp. isolates from clinical urine samples to nitrofurantoin and linezolid were 65% and 100%, respectively [9].

Due to the limited number of studies comparing different treatment options for UTIs caused by *Enterococcus* spp., this study aimed to evaluate the outcomes of the two major *Enterococcus* species, assess the outcomes of different antibiotics, and identify predictors associated with clinical cure and other clinical outcomes.

## 2. Materials and Methods

### 2.1. Study Design

This retrospective multicenter cohort study included adult patients who were admitted to or visited an outpatient clinic at King Abdulaziz University Hospital in Jeddah, Saudi Arabia, King Abdullah Medical City in Makkah, Saudi Arabia, and Sultan Qaboos University Hospital in Muscat, Oman, between June 2012 and December 2022, had clinically and microbiologically confirmed enterococcal UTI, and received antibiotic therapy. All patients who were available during the study period were pre-screened for eligibility using the electronic medical record system.

### 2.2. Study Population

The inclusion criteria for the study were patients aged 18 years or older who had a urine culture positive for *E. faecalis* or *E. faecium* with a colony count of ≥10^5^ CFU/mL, with symptoms of upper or lower urinary tract infection (fever, flank or back pain, nausea and/or frequency, dysuria, urgency, nocturia, and/or suprapubic pain), and received definitive antibiotic therapy targeting *Enterococci* as outpatients, during hospitalization, or upon discharge from the hospital. The cutoff value of bacterial load of 10^5^ CFU/mL was selected based on an established microbial threshold for bacteriuria [10]. Patients with incomplete data and those who received meropenem for therapy were excluded as meropenem has weak in vitro activity against *Enterococci* [11]. Only the first infection was considered in patients with recurrent enterococcal UTI.

All microbiological methods for bacterial culturing followed the guidelines of Clinical Laboratory Standards Institute (CLSI) [12]. Bacterial identification was carried out using matrix-assisted laser desorption/ionization time-of-flight (MALDI-TOF) mass spectrometry (by bioMérieux, Marcy-l’Étoile, France, in King Abdulaziz University Hospital and by Bruker Corp., Billerica, MA, USA, in King Abdullah Medical City and Sultan Qaboos University Hospital). This was followed by antibiotic susceptibility testing that was conducted using automated methods, including VITEK2 (bioMérieux, Marcy-l’Étoile, France) in King Abdulaziz University Hospital and King Abdullah Medical City and BD Phoenix™ (Franklin Lakes, NJ, USA) in Sultan Qaboos University Hospital. Minimum inhibitory concentration (MIC) interpretation in all participating hospitals followed CLSI guidelines (document M-100-S32), where *Enterococcus* spp. were considered resistant at or above the following MIC values: 16 µg/mL for ampicillin, 32 µg/mL for vancomycin, 8 µg/mL for levofloxacin, and 128 µg/mL for nitrofurantoin [12]. These MIC breakpoints did not change over the study period (2012–2022).

### 2.3. Clinical Outcomes and Definitions

The primary outcomes included clinical cure (defined as the resolution of signs and symptoms of UTI at the end of therapy). As such, treatment failure involved persistence of symptoms requiring a change in antibiotic therapy or death. For outpatients, this was assumed if patients did not return complaining of a lack of improvement or a recurrence. Microbiological cure was defined as the eradication of initial bacteriuria in the follow-up urine culture if available. The secondary outcomes included in-hospital mortality, defined as death during hospitalization for inpatients (as such, death was not recorded for outpatients). The length of hospital stay (LOS) was defined as the number of days from admission to discharge for surviving hospitalized patients. Recurrence was categorized as either relapse or reinfection. Relapse was defined as infection with the same organism isolated in the index culture within two weeks after therapy completion, whereas reinfection was defined as infection with an organism different from that of the index culture within or after two weeks of therapy completion, infection with the same organism as in the index culture more than two weeks after therapy completion, or a sterile intervening culture (defined as lack of bacterial growth between two UTI episodes while the patient is off antibiotics and is asymptomatic [i.e., after achieving microbiological cure]) [13,14]. Complicated UTI factors were defined by the presence of certain factors, such as structural or functional abnormality of the urinary tract, pregnancy, or prolonged symptoms, as described previously [15]. Polymicrobial cultures were defined as the presence of one or more organisms along with the *Enterococcus* strain that is considered pathogenic (i.e., not a contaminant, such as yeast and *Streptococcus agalactiae*) [16]. Examples of organisms that were considered potentially pathogenic include members of the Enterobacterales family (such as *Escherichia coli*, *Klebsiella pneumoniae*, *Proteus mirabilis*, etc.) and *Pseudomonas aeruginosa.*

Antibiotic therapy targeting *Enterococci* was administered intravenously or orally using one of the following agents: ampicillin (or amoxicillin), vancomycin, daptomycin, ciprofloxacin, piperacillin/tazobactam, linezolid, or nitrofurantoin.

### 2.4. Statistical Analysis

Patients were divided into two groups depending on the species (*E. faecalis* and *E. faecium* groups). Categorical variables were presented as frequencies and percentages and were compared using the Chi-square test or Fisher’s exact test (when the frequency was less than five times), while continuous variables were expressed using the median and interquartile range (IQR), as they were determined to be not normally distributed with the Shapiro–Wilk test of normality. The Mann–Whitney test was used to compare the continuous variables between the two groups. However, in the subgroup analyses, the Kruskal–Wallis test was used to compare the outcomes of each antibiotic against each species. The two-tailed significance threshold was set at *p* < 0.05.

To assess the impact of different factors on clinical outcomes, multivariable regression was performed on factors with *p* < 0.05 in the univariate analysis. Adjusted odds ratios (ORs) and 95% confidence intervals (95% CIs) were reported. Significance was assessed using the omnibus test of model coefficients, and the goodness-of-fit of the model was assessed using the Hosmer–Lemeshow test. All statistical analyses were performed using SPSS version 24.0 (IBM Corp., Armonk, NY, USA).

A total sample size of 263 patients was needed to achieve a power of 90% considering an effect size of 20% in the difference of clinical cure between the two Enterococcal species and an α-error probability of 5%. The effect size was determined based on a low probability estimate due to the lack of such studies in the literature. Sample size calculation was done using G*Power version 3.1.9.7 (University of Dusseldorf, Dusseldorf, Germany).

### 2.5. Ethical Declarations

This study was approved by the Institutional Review Board of King Abdullah Medical City (approval number: 23-1031; date 15 January 2023) and the Medical and Research Ethics Committee of the College of Medicine and Health Sciences, SQU, Muscat, Oman (approval number: MREC #3198; SQU-EC/321/2023; dated 9 January 2024). Informed consent was waived given the retrospective nature of the study.

## 3. Results

### 3.1. Baseline Characteristics

A total of 280 patients were included, with a median age of 62.5 years [IQR, 41.2–74.9], and more than half (57.1%) were female. *E. faecalis* and *E. faecium* were isolated from 188 (67.1%) and 92 (32.9%) patients, respectively. As listed in Table 1, most baseline characteristics were balanced between the two groups based on the species, except for patients’ location as outpatients or inpatients, distribution of the sites where *Enterococcus* spp. was isolated, and the antibiotic therapy used. Approximately one-third of the total cohort (*n* = 57; 32.2%) had secondary bacteremia. Among the 15 ICU patients, six (40%) had bacteremia, and two of them died. Additionally, more *E. faecium* isolates compared to *E. faecalis* isolates were resistant to ampicillin (82.6% vs. 8.5%), vancomycin (32.6% vs. 1.1%), and nitrofurantoin (52.2% vs. 1.6%%) with a *p* < 0.0001 for all comparisons. However, no difference was observed in rates of resistance to levofloxacin. The growth of organisms other than *Enterococci* was more prevalent with *E. faecium* than with *E. faecalis* (42.4% vs. 28.2%; *p* = 0.017).

### 3.2. Outcomes of Patients

Table 2 shows the clinical outcomes of enterococcal UTIs stratified by species. More than two-thirds of the patients in the overall cohort achieved clinical (69.3%) and microbiological cure (67.5%). For the latter, 169 of 280 patients had follow-up urine cultures (i.e., 111 patients did not have follow-up cultures). Both clinical cure and bacterial eradication rates were significantly higher with *E. faecalis* than with *E. faecium* (75% vs. 57.6% and 74.1% vs. 54.4%; *p* = 0.003 and 0.010, respectively). Moreover, patients infected with *E. faecalis* compared with *E. faecium* patients had lower in-hospital mortality rates (15.7% vs. 38.5%; *p* < 0.0001) and shorter median LOS (12.5 vs. 25 days; *p* < 0.0001). However, no difference was observed in recurrence or recurrence types (relapse or reinfection).

### 3.3. Subgroup Analysis Based on Enterococcal Species

In the subgroup analysis of patients with *E. faecalis* (Table 3), clinical cure rates were only numerically the highest with ciprofloxacin (*n* = 29/33; 87.9%), nitrofurantoin (*n* = 14/16; 87.5%), piperacillin/tazobactam (*n* = 16/21; 76.2%), and ampicillin or amoxicillin (*n* = 51/67; 76.1%), with a *p* value of 0.064. The median duration of therapy ranged from 6 to 8 days (*p* = 0.017). Statistically significant variations were observed between the antibiotics and in-hospital mortality and recurrence rates. In terms of *E. faecium* infections, all patients treated with ampicillin or amoxicillin (*n* = 3), piperacillin/tazobactam (*n* = 5), and ciprofloxacin (*n* = 4) achieved clinical cure, which was statistically significant with a *p* value of 0.012 (Table 4). While no difference was observed in microbiological cure, duration of therapy, and recurrence rates, a significant difference (*p* < 0.0001) was observed in the median LOS (the shortest duration was 0.5 days with ampicillin or amoxicillin and 6 days with ciprofloxacin) and in-hospital mortality (*p* = 0.019).

### 3.4. Factors Associated with Different Outcomes

The *p* values of the univariate analysis of the association of factors with each clinical outcome are provided in Table S1. Bacteremia was one of the evaluated factors that was not associated with any outcome; thus, it was not included in the multivariable analysis of all outcomes. Results of the multivariable logistic regression for clinical cure, microbiological cure, in-hospital mortality, and recurrence are presented in Table 5. The analysis revealed that the use of ciprofloxacin and having a risk factor for complicated UTI were associated with odds of clinical cure of 4.28 and 3.78 (95% CI, 1.18–15.56 and 1.33–10.77; *p* = 0.028 and 0.013, respectively). In contrast, recent exposure to cancer chemotherapy, the growth of *Enterococci* in cultures other than the urine culture (mostly bacteremia), and use of vancomycin for therapy were associated with lower odds of clinical cure (OR, 0.18, 0.41, and 0.46; 95% CI, 0.07–0.52, 0.17–0.97, and 0.22–0.98; *p* = 0.001, 0.042, and 0.044, respectively). While admission to the inpatient medical floor was associated with higher odds of bacterial eradication (OR, 3.27; 95% CI 1.18–9.08; *p* = 0.023), female sex, resistance to ampicillin, and polymicrobial culture were associated with lower odds of bacterial eradication (OR, 0.34, 0.31, and 0.43; 95% CI, 0.16–0.76, 0.10–0.97, and 0.20–0.93; *p* = 0.008, 0.044, and 0.032, respectively). Furthermore, the odds of in-hospital mortality increased with advancing age (OR, 1.03; 95% CI 1.01–1.04; *p* = 0.007) and admission to the ICU (OR, 5.55; 95% CI, 1.45–21.34; *p* = 0.013). Lastly, only removal of the urinary catheter was associated with lower odds of recurrence (OR, 0.48; 95% CI, 0.24–0.98; *p* = 0.044), whereas treatment with ampicillin was associated with higher odds of recurrence (OR, 2.94; 95% CI, 1.40–6.17; *p* = 0.005).

## 4. Discussion

This study offers critical insights into the clinical characteristics, resistance patterns, and treatment outcomes of enterococcal UTIs, with a particular focus on comparing the outcomes of infections with *E. faecalis* and *E. faecium*. The higher prevalence of *E. faecalis* in our study compared with *E. faecium* corroborates previous reports of enterococcal UTIs [17,18,19]. Our findings emphasized poorer clinical outcomes of UTI caused by *E. faecium*, which was previously reported in terms of lower clinical and microbiological cure rates, higher in-hospital mortality rates, and longer hospital stays [7]. However, the results of the multivariable regression analysis showed no difference in outcomes between the two species.

Our study revealed that the median age of patients with enterococcal UTIs was 63 years, a finding consistent with other studies, such as that by Kajihara et al., where the average patient age was 64.6 years [20]. However, a significant variability in age demographics was observed across studies. For example, Barros et al. reported a younger average age of 48.9 years in their cohort [21], while a multinational study by Turjeman et al. found that over 60% of patients were between 55 and 75 years old [18]. This suggests that the age distribution of enterococcal infections may vary significantly depending on population characteristics, healthcare settings, and geographical factors.

The gender distribution in our study also differed from some prior research. While several studies, including those by Barros et al. and Turjeman et al. reported a male predominance in enterococcal infections [18,21], our study found that 57.9% of the patients were female. This discrepancy can be explained by the higher susceptibility of females to UTIs due to anatomical factors, such as a shorter urethra and proximity of the urethral opening to the vaginal and rectal areas, facilitating bacterial translocation to the urinary tract. This is consistent with the general understanding that women are more prone to UTIs overall, although male predominance has been noted in specific populations with hospital-acquired infections or in patients with complex comorbidities.

A key finding of our study is the difference in clinical outcomes and resistance patterns between *E. faecalis* and *E. faecium* infections. The clinical cure rate was significantly higher with *E. faecalis* compared with *E. faecium*. On the contrary, the mortality rate was significantly lower with *E. faecalis* than with *E. faecium*. This is consistent with findings from a study by Álvarez-Artero et al., who found that the OR of mortality in the case of UTIs due to *E. faecalis* was 0.1 (95% CI, 0.1–0.3; *p* = 0.0001) [17]. The poorer outcomes associated with *E. faecium* observed in the current and previous studies may be attributed to the higher rates of resistance to commonly used antibiotics. For example, in our study, *E. faecium* exhibited a strikingly high resistance rate to ampicillin, moderate resistance to vancomycin, and moderate resistance to nitrofurantoin compared with *E. faecalis*, which exhibited very low resistance rates to these antibiotics. Such a resistance pattern by *E. faecium* makes the treatment of UTIs caused by this organism more challenging. This finding mirrors global trends, since *E. faecium* is known for its multidrug resistance, particularly in hospital settings, where vancomycin-resistant *E. faecium* poses a significant clinical challenge [22]. This underscores the importance of early identification and aggressive treatment of *E. faecium* infections, particularly in vulnerable populations. The findings related to antibiotic resistance, particularly the high resistance of *E. faecium* to ampicillin and vancomycin, reflect global concerns about the rising prevalence of VRE. Previous reports have highlighted the clinical and economic burdens posed by VRE, resulting in prolonged hospital stays, increased healthcare costs, and higher mortality rates [6]. The results of our study reinforce the urgent need for improved antimicrobial stewardship and infection control measures, particularly in healthcare settings where VRE is more prevalent. The rising resistance of *E. faecium* to key antibiotics, such as vancomycin, underscores the necessity for species-specific treatment strategies, as *E. faecalis* and *E. faecium* exhibit distinctly different resistance patterns and clinical outcomes. Another potential explanation for the high mortality rate in the *E. faecium* group is the numerically high rate of cardiovascular and kidney diseases among the patients.

In our study, in-hospital mortality was reported in 23.2% of hospitalized patients. Although the cause of mortality was not captured from the patients’ medical records in our study, we evaluated several factors for their potential association with this outcome. While 32.2% of the total cohort in our study had bacteremia, this factor was not significantly associated with mortality. Conversely, increasing age and ICU admission illustrated a significant association in the multivariable analysis. Our study also revealed that 40% of the ICU patients had secondary bacteremia, where two incidents were fatal. In a study by Suppli et al., hospitalization in the ICU was associated with 4.2 odds of 30-day all-cause mortality of patients with enterococcal bacteremia (95% CI, 1.7–10; *p* = 0.002) [23]. This could be attributed to the fact that many patients in the ICU tend to have multiple comorbidities and varying hemodynamics [24]. In addition, older age was found to be an independent predictor of mortality in patients with enterococcal bacteremia (hazards ratio, 1.02; *p* = 0.005) [25]. Notably, numerous previous studies have demonstrated that enterococcal bacteremia is associated with mortality rates exceeding 20% [23,25,26,27].

In terms of recurrence, a study by Salm et al. evaluated outpatient males and reported a recurrence rate of enterococcal UTI reaching 25.9% compared to 22.2% with *Escherichia coli*, a common cause of UTIs (*p* < 0.001) [28]. The reason behind the relatively high recurrence rate reported by Salm et al. and in our study could be explained from a microbiological perspective, where *Enterococci* have been shown to have the capacity to live within urothelial cells of the ascending urinary tract. Horsley et al. confirmed the shedding of the *E. faecalis* within the urothelial cells of patients with chronic UTIs [29]. Moreover, a study on a mouse model using *E. faecium* demonstrated the capacity of the organism to form biofilms on the urothelial surface of the urinary tract, which could also form on indwelling catheters [30]. This virulence factor may explain why urinary catheter removal was significantly associated with lower odds of recurrence in our study.

Our study also highlights important differences in the effectiveness of various antibiotics in treating *E. faecalis* and *E. faecium* infections. For *E. faecalis*, ciprofloxacin and nitrofurantoin (for cystitis only) were most associated with higher clinical cure rates. Previous studies, including those by Swaminathan and Alangaden, have also reported that *E. faecalis* is generally more susceptible to first-line antibiotics like nitrofurantoin and amoxicillin [4]. Conversely, among patients with *E. faecium* infections, treatment with ciprofloxacin and piperacillin/tazobactam was associated with favorable outcomes, with both achieving 100% clinical cure rates, whereas vancomycin treatment was associated with a high mortality rate. However, such results should be carefully interpreted given the small sample number of patients who received these three antibiotics (4, 5, and 29, respectively). Moreover, the effectiveness of newer agents, such as linezolid and daptomycin, especially for *E. faecium*, which had notable success in previous studies, should be considered as part of the therapeutic arsenal against this multidrug-resistant organism. Linezolid, in particular, has shown promise in treating VRE infections (clinical cure rate reaching 71.4% in our study); however, its use should be judicious to prevent the emergence of resistance. The variability in treatment outcomes between the two species, particularly the poor response to vancomycin by *E. faecium*, highlights the need for ongoing research into alternative therapeutic options and tailored antibiotic protocols based on local resistance patterns.

Several factors were evaluated for their potential association with major clinical outcomes, including clinical cure, microbiological cure, and in-hospital mortality. Two factors were positively associated with clinical cure, including ciprofloxacin use and the presence of a risk factor for a complicated UTI, which had approximately four times the odds of achieving clinical cure. Ciprofloxacin has long been known to be well excreted in the urine and was associated with good clinical outcomes in treating both upper and lower UTIs due to Gram-positive and Gram-negative organisms [31,32]. Moreover, in the subgroup analysis, patients who received ciprofloxacin also experienced shorter LOS than those who received its antibiotic counterparts. Nevertheless, only a few patients in the *E. faecium* group received ciprofloxacin compared with the number of patients in the *E. faecalis* group. This may explain the inflated OR that favors this agent for enterococcal UTI treatment. Notably, a review article spanning 10 years that evaluated antimicrobial resistance found that the average overall susceptibility rate of *E. faecalis* to ciprofloxacin was 67% [33].

Patients with a risk factor for a complicated UTI tended to receive antibiotics with high rates of clinical cure (>70%), such as piperacillin/tazobactam, ampicillin, and ciprofloxacin, which explains their positive association with clinical cure. The high odds of clinical cure associated with complicated UTI could be possibly explained by more extensive management, which may have included catheter removal (i.e., source control), since 59.3% of the total cohort had a urinary catheter at the time of UTI diagnosis, and about two-thirds (64.5%) had their catheter removed. Cancer chemotherapy within three months and growth of *Enterococcus* spp. in another non-urine sample were associated with negative odds of clinical cure. This can be explained by the immunosuppression state and a high rate of antimicrobial resistance in cancer patients as well as the isolation of *Enterococcus* from other sites, including the blood [34,35,36].

Liver disease and polymicrobial culture were negatively associated with bacterial eradication. This can be attributed to the use of broad-spectrum antibiotic therapy in polymicrobial infections, which are potentially less potent against *Enterococcus* spp. than other organisms, such as meropenem or cefepime. A previous study found that a high proportion of patients with liver disease had indwelling catheters inserted, which increased the risk of bacteriuria by 5–10% each day [37]. Interestingly, among the 25 patients with liver disease in our study, 15 (60%) had catheters inserted. In our study, a urine culture was considered polymicrobial only if the coexisting organism (or organisms) is (or are) potentially pathogenic and not commonly known as a urine contaminant, such as yeast or *Streptococcus agalactiae*, based on a consensus developed by Moreland et al. [16]. Furthermore, a comprehensive review by Gaston et al. concluded that the presence of multiple microbes in the urinary tract can facilitate dynamic intermicrobial interactions, such as enhanced mutagenesis and production of toxins or adhesins [38]. These microbial consortia can augment the virulence of the pathogens, resulting in increased disease severity.

Five factors were associated with more than one odds of in-hospital mortality: advanced age, ICU admission, liver disease, hemodialysis, and receiving vancomycin for treatment. Overall, patients with chronic liver disease and those on hemodialysis are known to have an increased risk of mortality due to their vulnerability to severe infections and the development of antibiotic resistance following frequent exposures to antibiotics [37,39,40]. The potential emergence of vancomycin resistance during therapy may have contributed to the high mortality observed in patients who received vancomycin. While this was not specifically evaluated in our study, it was assumed based on findings from previous clinical reports [41,42,43]. In our study, the overall mortality rate in patients who received vancomycin was 40.32% (25 of 62 patients).

Although our study provided important insights into the clinical characteristics and treatment outcomes of enterococcal UTIs considering the differences between the two major species, a few limitations should be considered when interpreting the results. The retrospective nature of the study resulted in the lack of some clinical data, such as follow-up microbiological culture results. Moreover, the heterogeneity of the patients being included from outpatients and inpatients, as well as including both upper and lower UTIs, should also warrant careful interpretation of the results. The reasons behind in-hospital mortality were not evaluated; thus, we could not count the number of patients who died of UTI, as some patients may have died due to other underlying comorbidities, especially considering the high rate of cardiovascular and kidney diseases in our patient cohort, where we found numerically high rates of mortality in patients with these conditions, as well as in patients with liver disease and cancer. Additionally, the previous history of recent antibiotic exposure was only documented based on data available in the medical records of patients. Hence, receiving antibiotics from other sources (such as primary healthcare clinics) could not be captured. Moreover, antibiotic susceptibility testing methods and antibiotic panels varied among the participating hospitals; thus, only the susceptibility patterns to the antibiotics commonly used in clinical practice by all three hospitals were reported in this study without reporting the exact MIC values, since two of the participating hospitals only include MIC interpretation in their culture and susceptibility reports. Furthermore, although the study included three hospitals from three large cities in Saudi Arabia and Oman, the results may not be generalizable to other hospitals in the Gulf region or to hospitals located in small cities or rural areas. Lastly, some patients had polymicrobial urinary cultures, where the coexisting pathogens may have had a potential influence on outcomes, though such patients were included in the study cohort to align with the patient population encountered in standard clinical practice.

## 5. Conclusions

This study underscores the clinical burden of enterococcal UTIs, particularly those caused by *E. faecium*. Although the findings revealed that patients infected with *E. faecium* had poorer clinical outcomes than those infected with *E. faecalis*, possibly due to higher rates of antibiotic resistance, the multivariable analysis demonstrated no difference in outcomes. Ciprofloxacin remains an effective option against *E. faecalis* in particular. It was also effective in the four patients who were infected with *E. faecium*. However, nitrofurantoin (for cystitis only), ampicillin or amoxicillin, and piperacillin/tazobactam were effective against *E. faecalis* only. On the other hand, ampicillin or amoxicillin, piperacillin/tazobactam, and linezolid were effective against *E. faecium* despite the small number of patients who received them. Of note, close monitoring should be considered for elderly patients, patients receiving chemotherapy, patients with complicated infections (i.e., those with a second culture from a different site growing *Enterococcus* spp.), critically ill patients in the ICU, patients with underlying liver disease, and patients on hemodialysis to improve their outcomes. Our findings suggest that vancomycin should be used cautiously in enterococcal UTIs, with repeat susceptibility testing in case of inadequate clinical response to assess the possible development of resistance during therapy. Similar attention should be given to ampicillin or amoxicillin in patients prone to recurrent infections. Finally, urinary catheter removal should be prioritized in patients with indwelling catheters to reduce the risk of recurrence. Future research is warranted to assess the incidence of antimicrobial resistance development by *Enterococci* during therapy and to elucidate the potential underlying mechanisms.

## Figures and Tables

**Table 1:** Baseline characteristics of patients with enterococcal urinary tract infections.

Characteristic *n* (%) Unless Specified Otherwise	All Patients (*n* = 280)	*Enterococcus faecalis* (*n* = 188)	*Enterococcus faecium* (*n* = 92)	*p* Value
Age (years); median [IQR]	62.5 [41.2–74.9]	61.9 [41.8–73.4]	65.3 [39.9–79.7]	0.212
Gender (female)	160 (57.1)	106 (56.4)	54 (58.7)	0.713
Location				
Outpatient	54 (19.3)	45 (23.9)	9 (9.8)	0.005
Non-ICU inpatient	211 (75.4)	133 (70.7)	78 (84.8)	0.010
ICU	15 (5.4)	10 (5.3)	5 (5.4)	0.968
UTI site				0.165
Upper/pyelonephritis	123 (43.9)	88 (46.8)	35 (38)	
Lower/cystitis	157 (56.1)	100 (53.2)	57 (62)	
Comorbidities				
Diabetes	144 (51.4)	100 (53.2)	44 (47.8)	0.399
Dyslipidemia	43 (15.4)	28 (14.9)	15 (16.3)	0.758
Thyroid disease	30 (10.7)	22 (11.7)	8 (8.7)	0.445
Kidney disease	133 (47.5)	87 (46.3)	46 (50)	0.558
Liver disease	22 (7.9)	15 (8)	7 (7.6)	0.914
Cardiovascular disease	188 (67.1)	123 (65.4)	65 (70.7)	0.382
Cancer	42 (15)	29 (15.4)	13 (14.1)	0.776
Solid organ transplant	9 (3.2)	4 (2.1)	5 (5.4)	0.141
Bone marrow transplant	1 (0.4)	0	1 (1.1)	0.152
Hemodialysis	24 (8.6)	12 (6.4)	12 (13)	0.086
Recent hospitalization *	129 (46.1)	85 (45.2)	44 (47.8)	0.680
Recent antibiotic use *	113 (40.4)	73 (38.8)	40 (43.5)	0.456
Recent chemotherapy *	25 (8.9)	17 (9)	8 (8.7)	0.924
Recent immunosuppressant use *	32 (11.4)	24 (12.8)	8 (8.7)	0.315
Having a risk factor for complicated UTI	256 (91.4)	174 (92.6)	82 (89.1)	0.337
Other risk factors for complicated UTI				0.488 **
Structural or functional abnormality of urinary tract	59 (21.1)	45 (23.9)	14 (15.2)	
Genitourinary obstruction	19 (6.8)	15 (8)	4 (4.3)	
Prolonged symptoms (>7 days)	19 (6.8)	12 (6.4)	7 (7.6)	
Pregnancy	7 (2.5)	6 (3.2)	1 (1.1)	
Nosocomial or nursing home-acquired infection	8 (2.3)	5 (2.7)	3 (3.3)	
Urinary catheter at baseline	166 (59.3)	104 (55.3)	62 (67.4)	0.053
Antibiotic resistance				
Ampicillin	92 (32.9)	16 (8.5)	76 (82.6)	<0.0001
Vancomycin	32 (11.5)	2 (1.1)	30 (32.6)	<0.0001
Nitrofurantoin	51 (18.2)	3 (1.6)	48 (52.2)	<0.0001
Levofloxacin	87 (31.1)	53 (28.2)	34 (37)	0.137
Polymicrobial culture	92 (32.9)	53 (28.2)	39 (42.4)	0.017
Other enterococcal culture	47 (16.9)	33 (17.7)	14 (15.2)	0.597
Other enterococcal culture site				0.044 **
Blood	57 (32.2)	44 (39.3)	13 (20)	
Respiratory	9 (5.1)	4 (3.6)	5 (7.7)	
Skin or soft tissue	2 (1.1)	0	2 (3.1)	
Body fluid	4 (2.3)	2 (1.8)	2 (3.1)	
Cerebrospinal fluid	1 (0.5)	1 (0.9)	0	
Urinary catheter removed (*n* = 166)	107 (64.5)	70 (67.3)	37 (59.7)	0.320
Antibiotic therapy				<0.0001 **
Ampicillin or amoxicillin	70 (25)	67 (35.6)	3 (3.3)	
Vancomycin	62 (22.1)	33 (17.6)	29 (31.5)	
Daptomycin	46 (16.4)	17 (9)	29 (31.5)	
Ciprofloxacin	37 (13.2)	33 (17.6)	4 (4.3)	
Piperacillin/tazobactam	26 (9.3)	21 (11.2)	5 (5.4)	
Linezolid	22 (7.9)	1 (0.5)	21 (22.8)	
Nitrofurantoin	17 (6.1)	16 (8.5)	1 (1.1)	
Duration of antibiotic therapy (days); median [IQR]	7 [5–9]	7 [5–8]	7 [5–10]	0.363

ICU, intensive care unit; IQR, interquartile range; UTI, urinary tract infection. * Within 3 months of presentation. ** The *p* value is for the collective comparison of all the items included in this subheading.

**Table 2:** Outcomes of patients with enterococcal urinary tract infections.

Outcome *n* (%) Unless Specified Otherwise	All Patients (*n* = 280)	*Enterococcus faecalis* (*n* = 188)	*Enterococcus faecium* (*n* = 92)	*p* Value
Clinical cure	194 (69.3)	141 (75)	53 (57.6)	0.003
Microbiological cure (*n* = 169) *	114 (67.5)	83 (74.1)	31 (54.4)	0.010
In-hospital mortality (*n* = 257) **	61 (23.7)	26 (15.7)	35 (38.5)	<0.0001
Length of stay (days); median [IQR] (*n* = 240)	15.5 [7–32]	12.5 [6–22]	25 [10–54]	<0.0001
Recurrence (*n* = 276)	88 (31.9)	62 (33.5)	26 (28.6)	0.408
Recurrence type				0.670
Relapse	30 (34.1)	22 (35.5)	8 (30.8)	
Reinfection	58 (65.9)	40 (64.5)	18 (69.2)	

IQR, interquartile range. * For patients with available follow-up urine cultures. ** Outpatients were excluded.

**Table 3:** Treatment outcomes of patients infected with *Enterococcus faecalis* (*n* = 188) *.

Outcome *n* (%) Unless Specified Otherwise	Ampicillin or Amoxicillin (*n* = 67)	Vancomycin (*n* = 33)	Nitrofurantoin (*n* = 16)	Piperacillin/Tazobactam (*n* = 21)	Ciprofloxacin (*n* = 33)	Daptomycin (*n* = 17)	*p* Value
Clinical cure	51 (76.1)	20 (60.6)	14 (87.5)	16 (76.2)	29 (87.9)	11 (64.7)	0.064
Microbiological cure (*n* = 112)	27 (69.2)	12 (63.2)	8 (88.9)	13 (86.7)	13 (81.3)	10 (71.4)	0.493
Duration of antibiotic therapy	8 [6–8]	8 [6–11]	8 [6–15]	6 [4–7.5]	7 [5–8]	7 [4–7.5]	0.017
In-hospital mortality (*n* = 166)	6 (10.7)	8 (25)	0 (0)	6 (28.6)	0 (0)	6 (35.3)	0.006
Length of stay (days); median [IQR] (*n* = 136)	11 [5.5–16.5]	20 [9–51]	23 [1.5–41.5]	12 [6–23]	9.5 [3–19.6]	14 [8.5–35]	0.060
Recurrence (*n* = 185)	31 (47.7)	5 (15.2)	4 (25)	3 (14.3)	10 (31.3)	9 (52.9)	0.005
Recurrence type							0.224
Relapse	11 (35.5)	4 (80)	1 (25)	1 (33.3)	4 (40)	1 (11.1)	
Reinfection	20 (64.5)	1 (20)	3 (75)	2 (66.7)	6 (60)	8 (88.9)	

IQR, interquartile range. * Of the 188 patients infected with *E. faecalis*, only one patient received linezolid, which was not included in the subgroup analysis.

**Table 4:** Treatment outcomes of patients infected with *Enterococcus faecium* (*n* = 92) *.

Outcome *n* (%) Unless Specified Otherwise	Ampicillin or Amoxicillin (*n* = 3)	Vancomycin (*n* = 29)	Linezolid (*n* = 21)	Piperacillin/Tazobactam (*n* = 5)	Ciprofloxacin (*n* = 4)	Daptomycin (*n* = 29)	*p* Value
Clinical cure	3 (100)	13 (44.8)	15 (71.4)	5 (100)	4 (100)	12 (41.4)	0.012
Microbiological cure (*n* = 57)	0 (0)	10 (62.5)	7 (53.8)	2 (66.7)	3 (100)	9 (45)	0.285
Duration of therapy	6; 4–7 **	7 [3.5–11]	8 [7.5–11]	8 [4.5–11.5]	5.5; 5–7 **	7 [5.5–10]	0.093
In-hospital mortality (*n* = 91)	0 (0)	17 (58.6)	5 (25)	0 (0)	0 (0)	13 (44.8)	0.019
Length of stay (days); median [IQR] (*n* = 82)	0.5; 0–1 **	23 [10.8–53.3]	72 [34–123.5]	16 [10–23.5]	6; 6–15 **	22 [7–47]	<0.0001
Recurrence (*n* = 91)	2 (66.7)	10 (34.5)	7 (33.3)	1 (25)	0 (0)	6 (20.7)	0.449
Recurrence type							0.238
Relapse	0 (0)	2 (20)	2 (28.6)	0 (0)	0 (0)	4 (66.7)	
Reinfection	2 (100)	8 (80)	5 (71.4)	1 (100)	0 (0)	2 (33.3)	

IQR, interquartile range. * Of the 92 patients infected with *E. faecium*, only one patient received nitrofurantoin, which was not included in the subgroup analysis. ** Due to the small sample size of inpatients, the median with the range is represented instead of the median [IQR].

**Table 5:** Evaluation of the association of different factors with clinical outcomes.

Factor	Adjusted Odds Ratio (95% Confidence Interval); *p* Value			
	Clinical Cure	Microbiological Cure	In-Hospital Mortality	Recurrence
Age	0.99 (0.97–1); 0.058	NA	1.03 (1.01–1.04); 0.007	NA
Gender (female)	NA	0.34 (0.16–0.76); 0.008	NA	1.65 (0.82–3.34); 0.160
Location				
Outpatient	NA	NA	NA	NA
Non-ICU inpatient	NA	3.27 (1.18–9.08); 0.023	NA	NA
ICU	NA	0.54 (0.08–3.47); 0.517	5.55 (1.45–21.34); 0.013	NA
UTI site		NA		NA
Upper/pyelonephritis	1.33 (0.67–2.64); 0.412	-	0.61 (0.26–1.43); 0.255	-
Lower/cystitis	Ref	-	Ref	-
Species				NA
*Enterococcus faecalis*	1.52 (0.61–3.80); 0.975	1.15 (0.38–3.52); 0.808	0.45 (0.14–1.45); 0.181	-
*E. faecium*	Ref	Ref	Ref	-
Liver disease	0.45 (0.15–1.36); 0.156	0.31 (0.08–1.15); 0.080	2.75 (0.77–9.80); 0.120	NA
Hemodialysis	NA	NA	2.06 (0.71–6.00); 0.185	NA
Recent antibiotic use	1.44 (0.74–2.82); 0.289	NA	NA	NA
Recent chemotherapy	0.18 (0.07–0.52); 0.001	NA	NA	NA
Having a risk factor for complicated UTI	3.78 (1.33–10.77); 0.013	NA	NA	NA
Antibiotic resistance				
Ampicillin	0.88 (0.35–2.20); 0.780	0.31 (0.10–0.97); 0.044	1.09 (0.36–3.26); 0.878	NA
Nitrofurantoin	NA	NA	1.02 (0.38–2.73); 0.964	NA
Levofloxacin	1.47 (0.69–3.15); 0.319	NA	0.68 (0.27–1.72); 0.410	NA
Polymicrobial culture	0.67 (0.32–1.37); 0.266	0.43 (0.20–0.93); 0.032	1.65 (0.80–3.43); 0.178	NA
Other enterococcal culture	0.41 (0.17–0.97); 0.042	NA	NA	NA
Urinary catheter removed	NA	NA	NA	0.48 (0.24–0.98); 0.044
Antibiotic				
Ampicillin or amoxicillin	NA	NA	0.53 (0.16–1.80); 0.312	2.94 (1.40–6.17); 0.005
Vancomycin	0.46 (0.22–0.98); 0.044	NA	3.40 (1.09–10.59); 0.035	NA
Nitrofurantoin	NA	NA	*	NA
Linezolid	NA	NA	1.85 (0.68–5.06); 0.232	NA
Piperacillin/tazobactam	NA	NA	2.62 (0.68–10.11); 0.162	NA
Ciprofloxacin	4.28 (1.18–15.56); 0.028	NA	*	NA
Daptomycin	0.57 (0.21–1.53); 0.268	NA	1.68 (0.45–6.20); 0.438	NA

ICU, intensive care unit; NA, not applicable (not included in the multivariable regression analysis because the *p* value in the univariate analysis was >0.05); UTI, urinary tract infection. * Odds ratio could not be computed due to having a value of zero in the number of patients who died (i.e., none of the patients who received nitrofurantoin or ciprofloxacin died).

## Data Availability

Data associated with this study are available from the corresponding author upon request.

## Supplementary Materials

The following supporting information can be downloaded at https://www.mdpi.com/article/10.3390/pathogens15030250/s1. Table S1 (*p* values of univariate analysis of the association of different factors with clinical outcomes) on the results of the univariate analysis is included in the Supplementary Materials.

## Abbreviations

The following abbreviations are used in this manuscript:

CFI	Colony-forming unit
CI	Confidence interval
ICU	Intensive care unit
IQR	Interquartile range
LOS	Length of stay
OR	Odds ratio
UTI	Urinary tract infection
VRE	Vancomycin-resistant *Enterococci*
