# Supplementary material for: Antibiotic Outcomes of Enterococcal Urinary Tract Infections: A Retrospective Analysis from Saudi Arabia and Oman
Source: Pathogens. 2026 Feb 26;15(3):250. doi: 10.3390/pathogens15030250 (PMC13028684; doi:10.3390/pathogens15030250)
Supplement: Supplementary file 1 [file pathogens-15-00250-s001.zip › pathogens-4165085-supplementary.pdf]

**Table S1.** *P* values of univariate analysis of the association of different factors with clinical outcomes.

| Factor | Clinical Cure | Microbiological Cure | In-hospital Mortality | Recurrence |
|---|---|---|---|---|
| Age | **0.002** | 0.091 | **<0.0001** | 0.101 |
| Gender (Female) | 0.125 | **0.042** | 0.902 | **0.019** |
| Location | | | | |
| Outpatient | 0.892 | 0.303 | 0.131 | 0.491 |
| Non-ICU inpatient | 0.252 | **0.029** | 0.426 | 0.924 |
| ICU | 0.051 | **0.025** | **0.001** | 0.310 |
| UTI site (Upper vs. Lower) | **0.022** | 0.560 | **0.009** | 0.650 |
| Species (*E. faecalis* vs. *E. faecium*) | **0.003** | **0.010** | **<0.0001** | 0.408 |
| Comorbidities | | | | |
| Diabetes | 0.953 | 0.590 | 0.726 | 0.534 |
| Dyslipidemia | 0.776 | 0.506 | 0.311 | 0.646 |
| Thyroid disease | 0.742 | 0.345 | 0.447 | 0.516 |
| Kidney disease | 0.282 | 0.997 | 0.052 | 0.151 |
| Liver disease | **0.012** | **0.020** | **0.048** | 0.994 |
| Cardiovascular disease | 0.729 | 0.365 | 0.276 | 0.363 |
| Cancer | 0.137 | 0.924 | 0.543 | 0.617 |
| Solid organ transplant | 0.575 | 0.280 | 0.088 | 0.411 |
| Bone marrow transplant | 0.132 | * | 0.072 | 0.493 |
| Hemodialysis | 0.771 | 0.611 | **0.004** | 0.224 |
| Recent hospitalization | 0.496 | 0.509 | 0.555 | 0.131 |
| Recent antibiotic use | **0.042** | 0.208 | 0.233 | 0.082 |
| Recent chemotherapy | **0.001** | 0.205 | 0.511 | 0.643 |
| Recent immunosuppressant use | 0.944 | 0.623 | 0.086 | 0.202 |
| Having a risk factor for complicated UTI | **0.009** | 0.072 | 0.145 | 0.449 |
| Urinary catheter at baseline | 0.997 | 0.160 | 0.063 | 0.714 |
| Antibiotic resistance | | | | |
| Ampicillin | **0.007** | **0.008** | **<0.0001** | 0.715 |
| Vancomycin | 0.202 | 0.076 | 0.468 | 0.759 |
| Nitrofurantoin | 0.073 | 0.069 | **0.030** | 0.675 |
| Levofloxacin | **0.031** | 0.092 | **0.032** | 0.275 |
| Polymicrobial culture | **<0.0001** | **0.006** | **<0.0001** | 0.585 |
| Other enterococcal culture | **0.010** | 0.202 | 0.762 | 0.979 |
| Bacteremia | 0.423 | 0.602 | 0.589 | 0.792 |

| | | | | |
|---|---|---|---|---|
| Catheter removed | 0.965 | 0.908 | 0.637 | **0.049** |
| Antibiotic used | **<0.0001** | 0.127 | **<0.0001** | **0.028** |
| Duration of antibiotic therapy | 0.110 | 0.709 | 0.059 | 0.833 |

ICU, intensive care unit

* Odds ratio could not be computed due to having a value of zero in the number of patients who underwent bone marrow transplant and had a follow-up culture (i.e., none of the patients who had a follow-up culture underwent bone marrow transplant).
